# Analysis of transfusions records among thalassemia patients in Nellore, India

**DOI:** 10.6026/973206300213881

**Published:** 2025-10-31

**Authors:** Sarangapani Seetha, Aravind A, Kshithija Balaji Tandri, Balaji T.S, Bhargavi K, Haritha C

**Affiliations:** 1 Department of Oral and Maxillo Facial Surgery, Narayana Dental College and Hospital, Chinta Reddy Palem Nellore, Andhra Pradesh, India; 2 Department of Medicine, Government Kilpauk Medical College, Chennai; 3 Department of medicine, S.V. Medical Colleges, Tirupathi, India; 4 Private Practitioner, Department of Dentistry, Balaji Dental clinic Nellore, Andhra Pradesh, India; 5 Department of Cardiology, Private practice, Aravind kidney Hospital Nellore, Andhra Pradesh, India; 6 Department of Medical oncology, Private practice, Apollo Hospital Nellore, Andhra Pradesh, India

**Keywords:** Thalassemia, blood transfusion, packed cells, bone marrow transplant, IRCS Nellore, transfusion trends

## Abstract

Thalassemia, a chronic transfusion-dependent disorder, poses significant challenges in ensuring consistent blood supply and long-term patient management. Therefore, it is of interest to analyze transfusion records from the IRCS Nellore Blood Center between January 2019 and May 2025. Hence, a total of 8,386 transfusions were recorded, including 6,546 packed cells and 698 platelets, with a steady annual rise peaking in 2024. Fourteen patients underwent or were referred for bone marrow transplantation across major centres. Thus, the increasing transfusion demand highlights the need for strengthened donor programs and expanded BMT facilities to improve thalassemia care in the region.

## Background:

Thalassemia comprises inherited hemoglobin disorders marked by ineffective erythropoiesis and chronic hemolytic anemia, leading to lifelong dependence on blood transfusions in severe cases such as β-thalassemia major. Globally, thalassemia affects about 1.5% of the population, with over 200,000 births annually, primarily in South and Southeast Asia, the Middle East, and the Mediterranean regions [1, 2]. India contributes significantly to this burden, with approximately 10,000-15,000 new thalassemia major cases each year and an estimated 35-45 million carriers [3, 4]. Regular blood transfusion remains the cornerstone of management for transfusion-dependent thalassemia (TDT), helping maintain hemoglobin above 9-10 g/dL and suppress ineffective erythropoiesis. However, frequent transfusions predispose patients to iron overload, alloimmunization, and transfusion-transmitted infections, necessitating vigilant monitoring and chelation therapy [5, 6]. Curative approaches such as bone marrow transplantation (BMT) and gene therapy have shown promise but remain inaccessible to many due to financial and infrastructural constraints in low-resource settings [7, 8]. In this scenario, regional blood transfusion centers play a pivotal role in comprehensive thalassemia care. The Indian Red Cross Society (IRCS), Nellore District Branch, actively supports thalassemia management through packed cell and platelet transfusions, patient registries, and BMT referrals. Maintaining optimal pre-transfusion hemoglobin levels is vital to prevent skeletal deformities and growth retardation, yet chronic transfusions contribute to progressive iron accumulation in vital organs, increasing morbidity and mortality if inadequately chelated [5]. Common chelators such as deferoxamine, deferiprone, and deferasirox offer effective control but require individualized administration and compliance [6]. Alloimmunization rates among multi-transfused patients range from 5%-30%, underscoring the need for extended phenotype matching and improved donor compatibility in multiethnic populations [7]. Moreover, despite rigorous screening transfusion-transmitted infections like hepatitis B, hepatitis C, and HIV persist, highlighting the importance of nucleic acid testing (NAT) and robust hemovigilance systems [7]. BMT remains the only definitive cure, with success rates exceeding 85% among low-risk patients at centers such as Sankalp India Foundation and CMC Vellore, though accessibility remains limited by donor shortages and costs [8]. Government and NGO initiatives have begun expanding support for curative care, yet transfusion and chelation remain mainstays in most cases. Given these realities, decentralized management through district-level institutions such as IRCS Nellore is essential to ensure equitable care, patient education, chelation compliance, and timely BMT referrals. Longitudinal monitoring of transfusion patterns and patient registrations at such centers provides valuable insights for optimizing health resource allocation and improving outcomes in thalassemia management. Therefore, it is of interest to evaluate seven-year trends (2019-2025) in patient registration and transfusion services at IRCS Nellore, offering a regional perspective on evolving thalassemia care demands.

##  Materials and Methods:

## Study design and setting:

A retrospective observational study was conducted using secondary data obtained from the Indian Red Cross Society (IRCS), SPSR Nellore District Branch, Andhra Pradesh. The study covered a seven-year period from January 2019 to May 2025 and aimed to evaluate the trends in thalassemia patient registration and transfusion services.

## Data collection:

Transfusion records were obtained from the IRCS Blood Center's monthly and annual reports. The dataset included the number of packed red blood cell (PC) units and platelet (PLT) units issued specifically to thalassemia patients each month. In addition, a registry of patients who underwent or were undergoing bone marrow transplantation (BMT) was reviewed to gather details such as patient name, address, socioeconomic status, and the healthcare institution that performed the BMT.

## Inclusion and exclusion criteria:

All patients with a confirmed diagnosis of thalassemia who were registered at the IRCS Nellore Blood Center and received transfusion support during the study period were included. Patients with incomplete transfusion records or those receiving transfusions for other hematologic conditions were excluded from the analysis.

## Data management and analysis:

The data were entered into Microsoft Excel and analyzed using descriptive statistics. Monthly and annual totals of PC and PLT transfusions were tabulated to identify trends over the years. The frequency of transfusions and number of patients referred for BMT were also documented. Visual representations such as graphs and tables were created to illustrate year-wise variations in transfusion services.

## Ethical considerations:

Approval for this study was obtained from the Indian Red Cross Society, SPSR Nellore District Branch. The study adhered to the ethical principles outlined in the Declaration of Helsinki. No personal identifiers were disclosed in the analysis or reporting to maintain confidentiality of the patients.

## Results:

A total of 8,386 transfusions were administered to thalassemia patients at IRCS Nellore between January 2019 and May 2025. The transfusions included packed red blood cell (PC) units and platelet (PLT) units. The number of transfusions showed a progressive increase over the years, reflecting a rising demand for blood products. The numbers of PC and PLT units issued annually are shown in Table 1 (see PDF). There was a significant rise in PC usage from 445 units in 2019 to 1,367 units in 2024. The highest total transfusions were recorded in 2024 (1,528 units). The month-wise transfusion pattern for selected years is presented in Table 2 (see PDF). Peak transfusion periods were observed from March to May and October to December across most years, likely due to seasonal disease exacerbations and scheduled BMT procedures. A total of 14 thalassemia patients from Nellore were referred for BMT. Their treatment centers and socioeconomic profiles are listed in Table 3 (see PDF). Of these, several had completed BMT while others remained under follow-up. The proportion of packed cells versus platelets given over the study period is displayed in Table 4 (see PDF). Packed cells accounted for the vast majority (78.1%) of total transfusions, highlighting their critical role in thalassemia management. These findings collectively reflect a substantial increase in transfusion needs over the years and demonstrate the crucial role of IRCS Nellore in delivering continuous transfusion support to thalassemia patients. The data also underscore the importance of facilitating BMT access to reduce transfusion dependency in eligible individuals.

## Discussion:

The present study provides a comprehensive overview of transfusion trends among thalassemia patients registered at the Indian Red Cross Society (IRCS), Nellore, over a seven-year period. The data reflect a steady and significant increase in packed red blood cell (PC) and platelet (PLT) transfusions, consistent with national trends showing rising thalassemia burden and improved access to transfusion services in India [1, 2]. The annual packed cell transfusion count rose from 445 units in 2019 to 1,367 units in 2024, indicating either an increase in patient load or frequency of transfusions per individual-both markers of expanding service delivery and clinical need. The increase in transfusion volumes aligns with earlier findings from regional hemovigilance programs in India, which have reported improved thalassemia diagnosis and treatment-seeking behavior in recent years [3, 4]. Several contributing factors may include heightened community awareness, institutional support for blood safety, and wider availability of chelation therapy, allowing patients to live longer and require ongoing transfusions [5, 6]. However, this rise also signals pressure on blood supply chains, necessitating proactive donor recruitment and component inventory management at district-level blood banks [7]. Monthly distribution trends revealed seasonal peaks in transfusions, notably between March-May and October-December. These patterns may correspond to climatic stressors or institutional scheduling of pre-transplant optimization and BMT preparations [8]. Prior studies have observed that thalassemia patients often experience worsening clinical parameters in certain months due to infections or hemolytic crises, further exacerbating transfusion needs [9, 10]. This underscores the need for anticipatory resource planning and preventive health services, especially in semi-urban settings like Nellore. The BMT referral data highlight the proactive role of IRCS Nellore in facilitating curative interventions for thalassemia. Bone marrow transplantation remains the only definitive cure, with optimal outcomes in patients younger than 10 years and with low transfusion exposure prior to transplant [11]. Although curative therapy is costly, government schemes and NGO collaborations-such as with Sankalp India Foundation and CMC Vellore-have improved access for children from low-income families [12, 13]. However, BMT accessibility is still limited in many rural areas due to donor unavailability and logistical challenges [14]. Thus, while transfusion support is essential, a dual approach incorporating both chronic management and curative pathways should be emphasized. Additionally, the proportion of platelet transfusions was relatively low compared to packed cells. This is consistent with standard clinical protocols, where PLT support is used selectively during acute bleeding episodes or pre-surgical optimization [15]. Maintaining strict transfusion thresholds, avoiding overuse, and adhering to national guidelines are vital to prevent alloimmunization and iron overload-two major complications in chronically transfused individuals.

## Conclusion:

IRCS Nellore's data highlights a growing demand for transfusion services among thalassemia patients and the center's evolving role in providing both supportive and referral-based care. Strengthening local blood donation networks, expanding chelation monitoring, and facilitating access to BMT can collectively improve long-term outcomes for these patients. Moreover, digital registries and integrated care models may help in tracking patient outcomes and resource utilization more effectively.
